# The Queue System at Hospitals

**Published:** 1899-04-15

**Authors:** 


### The Institutional Workshop.

### THE QUEUE SYSTEM AT HOSPITALS.

Considerable indignation has recently been expressed in Birmingham in regard to the system which seems to be in vogue at some of the hospitals there of keeping the out-patients waiting in the streets until the arrival of the appointed hour for opening the hospital doors. Mr. Councillor Malins, writing to one of the local papers, says, "Months ago I wrote to the committee of the Children's Hospital, pointing out the pitiful sight to be seen, when from half a dozen to fifty poor mothers, nearly every one with a sick child in her arms, could be seen waiting for an hour in the rain or snow, while the secretary was inside and would not open the door. I received a reply that the committee had thought long and anxiously about it, but were unable to arrange to open the door before the fixed time." Of course the patients are in the wrong. Of course they should not come earlier than the time stated on their tickets. Of course, if they choose to break the rules they know what to expect, and so on. All this is obvious enough, but we fancy that so long as hospitals go on the system of "first come first served," and arrange that the sum total of the waiting shall be very much less for the lucky first ones than for the later arrivals, so long will there be a queue outside the hospital doors. Naturally the indignant bystander thinks that all that is wanted is to open the doors a little earlier. That, however, is only a very partial remedy for the evil. We must look deeper both for the cause and the cure of the queue system. Except the few who happen to come in from the country by an ill-chosen train, or the new patients who act in ignorance, no one would choose to stand outside in the rain, or to sit on "icy steps," unless he looked for some great countervailing advantage. Usually this advantage is that the first comer is the first seen, and thus is also the first to get away, and there is no doubt that the root of the evil lies in the enormous amount of time that patients are too often made to waste in hospital out-patient departments, and this is the outcome of the staff not being sufficiently large for the number of patients to be seen. If hospitals would arrange to lessen the amount of time wasted in their waiting-rooms we may be sure that patients would not wait in the snow for the first chance of seeing the doctor.

